# Conservative Medicine

**Published:** 1861-11

**Authors:** Austin Flint

**Affiliations:** Prof. of the Principles and Practice of Medicine in the Bellevue Hospital Medical College, N. Y., and in the Long Island College Hospital


ART. I.—*Conservative Medicine.* By AUSTIN FLINT, M.D., Prof. of the Principles and Practice of Medicine in the Bellevue Hospital Medical College, N. Y., and in the Long Island College Hospital.

"WHAT does the writer mean by *Conservative Medicine?*" This will be the mental inquiry of the reader when the caption of this article meets his eye. It is desirable, first of all, for the writer to explain the subject which he ventures to hope will appear to possess interest enough to lead to a perusal of the pages which are to follow.

The meaning of *Conservative Surgery* is well understood. This phrase has been sufficiently common of late years. The conservative surgeon aims to preserve the integrity of the body. He spares diseased or wounded members whenever there are good grounds for believing that by skillful management they may be saved. He resorts to mutilations only when they are clearly necessary. He weighs carefully the dangers of operations, so as not to incur too much risk of shortening life by resorting to the scalpel. By conservative medicine, I mean an analogous line of conduct in the management of maladies which are not surgical. The conservative physician shrinks from employing potential remedies whenever there are good grounds for believing that diseases will pursue a favorable course without active interference. He resorts to therapeutical measures which must be hurtful if not useful, only when they are clearly indicated. He appreciates injurious medication, and hence does not run a risk of shortening life by adding dangers of treatment to those of disease. Such, in brief, is an explanation of the subject of this article; to give a fuller exposition of the subject is, in part, the purpose of the article. For the phrase *conservative medicine* I am indebted to a distinguished friend and colleague, well known as eminently a conservative surgeon.*

During the last quarter of a century a change has taken place in medical sentiment as regards surgical operations. New and grand achievements in surgery seemed formerly to be the leading objects of personal ambition. To borrow a fashionable expression, they were decidedly the rage. Boldness in the use of the knife was the trait in the character of the surgeon which was most highly admired. The history of surgery during the first third of the present century is characterized by the introduction and frequent per-

---

* Prof. Hamilton.

formance of numerous formidable operations. It was customary to speak of them as brilliant, and the daring surgeon enjoyed somewhat of the *éclat* which belongs to the hero of the battle-field. This analogy was implied when one of the greatest of our American surgeons, wishing to distinguish his most brilliant exploit, styled it his Waterloo operation. The change that has taken place is marked. We hear now comparatively little of terrible operations, and of that sort of heroism which is associated with bloody deeds. What would once have been considered as a degree of courage to be admired, is now stigmatized as rashness. It is an equivocal compliment to say of a practitioner that he is a bold surgeon. The change, it may be said, is in a measure due to the fact that the great number of new operations which have been introduced since the beginning of the present century leaves but a limited range for further explorations in that direction; but this explanation will go only a little way. The change is one of sentiment. The desire is to preserve the integrity of the body, to avoid mutilations, to incur the dangers of capital operations only when they are imperatively called for—in a word, conservatism has become the ruling principle in surgery. The most important of the more recent improvements in surgery exemplify the influence of this principle on the medical mind.

An analogous change, within the same period, has taken place in medical practice. Formerly, boldness was a distinction coveted by the medical, as well as by the surgical practitioner. "Heroic practice" was a favorite expression, consisting in the employment of powerful remedies, or in pushing them to an enormous extent. The physician emulated the surgeon in daring. The change is not less marked in medicine than in surgery. We hear now oftener of diseases managed with little or no medication, than of cases illustrating the abuse of remedies. In the treatment of many affections it is not considered necessary to employ measures which, but a few years ago, it would have been considered culpable to withhold. The change, too, is here one of sentiment. We desire to preserve the vital forces, to avoid the perturbations and damaging effects of potential therapeutic agencies—in short, conservatism has become a leading principle in medicine as well as in surgery. The improved method of managing a host of affections will be found to illustrate this fact.

Before proceeding further, let us inquire how the contrast between medical practice at the present moment and a quarter of a century ago, should affect our estimation of medicine Is medicine disparaged by the changes which have actually taken place? It is not enough to answer this question in the negative. Mutations, when they denote progress, are, of course, desirable. In so far as the contrast shows improvement, medicine at the present moment is deserving of esteem, the more, as the changes are great. It redounds to the glory of medicine that it admits of illimitable progressive changes. In this fact lies the distinctive feature of legitimate medicine as contrasted with illegitimate systems of practice. But, some one may say, is there to be no stability in medicine, no traditional authority, and is reverence for the past to have no influence? If not, where is our ground of confidence in the practice of the present day? And is it not probable that at the end of another quarter of a century mutations will have occurred quite as great as those which have taken place during the last twenty-five years? These questions are to be met fairly and squarely; let us endeavor so to meet them.

Waiving the consideration of what constitutes perfectibility in the *ars medendi*, and whether it be obtainable or not, no one will assert that medicine is now, or ever has been, in a condition not to admit of indefinite improvement. Improvement in its practical applications and results is the great end of the labors devoted, now and hitherto, to the different departments of medical knowledge, viz., anatomy, physiology, animal chemistry, materia medica, pathology, and clinical medicine. We may assume that these labors, thus far, have not been profitless, and, accordingly, that practical medicine has improved. We may assume, also, that there is abundant encouragement to continue these labors, and, hence, that further improvement is to be expected—to what extent it is vain to speculate. It necessarily follows that stability in medicine is not to be counted upon; that the doctrines of to-day have no intrinsic claim to perpetuity; that because they are now in vogue is not a sufficient reason why they should not hereafter be modified or rejected, and that there is, to say the least, no ground to deny the possibility of the changes which are to take place hereafter being a whit less than those which have already taken place. What then? There are skeptics and scoffers with regard to medicine, and there are many persons who live and thrive by promoting popular distrust of it. It may seem to be giving aid and comfort to the enemies of medicine to concede that its past history abounds in errors, that present errors doubtless abound, leaving ample scope for future improvement. Be it so. We have nothing to do with skeptics, scoffers, and charlatans. We are not called upon to repel attacks prompted by ignorance, selfishness, and deceit. Yet it is desirable, with regard not only to the interests of the medical profession, but to the welfare of humanity, that medicine should hold its proper place in popular estimation. What, then, is the attitude to be taken as regards the just claims of medicine to public consideration and confidence? A body of men in every generation, from the time of Hippocrates to the present day, in all civilized countries, have conscientiously and industriously labored to acquire knowledge of diseases with reference to the relief of suffering and the prolongation of life.

Under a host of difficulties and obstructions, many inherent in the pursuits themselves, and others proceeding from various extrinsic sources, the labors of physicians and their collaborators have continued and still continue. Now, granting that they have advanced slowly and often been led astray, where else can society ever seek for aid in the necessities of illness with a better prospect of success? Granting that they have failed, and still fail, in conferring all the benefit that is to be desired, and that, with the purest intentions, their efforts have been sometimes not only without avail, but hurtful, should the preponderating good be therefore overlooked, and is there any rational alternative but to accept good and submit to the limitations and errors incident to existing knowledge? All that society can claim of medicine in any generation is, the capabilities of the medical science in that generation. All that society can claim of physicians is, that these capabilities shall be understood and judiciously applied. But we are opening up trains of thought which will lead us a long way from our subject, and we must abruptly return to the consideration of conservative medicine.

It is an interesting point of inquiry, whence came the influences leading to conservatism as a principle of medical practice? The answer to this inquiry would not be the same in all countries and sections. It must be admitted that in our country the earliest and fullest development of the principle was in New England. Our New England brethren are fond of dating a new order of medical ideas from the publication of an address, more than twenty years ago, by Jacob Bigelow, on the self-limited character of certain diseases. Not underrating the importance of that publication, the spirit of the oral teachings of James Jackson and John Ware has exerted on the medical mind of New England an influence which can only be appreciated by those who have experienced it. To those who have known experimentally the value of their teachings, it is a source of deep regret that the influence of these admirable professors has not been more widely diffused by means of larger contributions to medical literature. British conservatists attribute much to the writings of Dr. Forbes. Among the non-medical observers of the change in practice which has taken place, some have been persuaded that it is due to the disciples of Hahnemann, an idea too preposterous to need refutation. The truth is, we are not to look for the causes of the change exclusively in the views emanating from particular persons. It is rather a legitimate result of scientific researches in different directions. If we were to specify circumstances which have more especially been instrumental in leading to the principle of conservatism, we would mention, *first*, the abandonment of the attempt to found a system or theory of medicine after the decline and fall of Brunonianism and Broussaisism; and *second*, the study of diseases after the numerical method with reference to their natural history and laws.

Strange as it appears, the importance of determining by clinical observation the intrinsic tendencies of different diseases as the basis of therapeutics, seems to have been heretofore overlooked. Physicians have acted on the presumption that most diseases do not pursue a favorable course without treatment more or less efficient. This has been, to a still greater extent, the popular belief. The apparent proof of the success of the Hahnemannic treatment rests on this belief. What are the facts already ascertained with respect to the intrinsic tendencies of different diseases? We know that diseases in the management of which, but a few years ago, the physician would not dare to omit potent therapeutical measures, almost invariably end in recovery without any active treatment. Take, as examples, pneumonia limited to a single lobe, and acute pleurisy. It is sufficiently settled that these diseases involve very little danger in themselves, proving fatal only in consequence of complications. The practitioner, therefore, no longer feels obliged to employ blood-letting, mercurialization, cathartics, blisters, etc., in these diseases, with reference to the saving of life. The only question is, do patients pass through these diseases as well without as with such measures of treatment? Clinical observation, following up this inquiry, arrives at results which exemplify conservative medicine.

Our acquaintance with the natural history of the great majority of diseases is, as yet, very incomplete. Knowledge of the tendencies of diseases allowed to pursue their course without active treatment, is not readily acquired. We cannot conscientiously withhold remedies which we have reason to believe may prove useful. Cases are therefore to be slowly accumulated in which, from circumstances not under our control, diseases have been uninfluenced by therapeutic interference. This knowledge, it is evident, is the true point of departure for the study of the effects of remedies as regards the termination and duration of diseases. The information already obtained has rendered the use of powerful therapeutic agencies far less common than they were but a few years since. It remains to be seen hereafter what will be the further effect on medical practice of continued researches in this direction.

Conservative medicine assumes that remedial measures, according to their potency, must either do harm or good; that they can never be neither hurtful nor useful. Prior to the advent of conservatism, this important fact was not duly appreciated. Blows were leveled at diseases, but the patient was not enough considered. It did not enter sufficiently into the calculations of practitioners that if successive blows dealt at a disease were misdirected, the effect was not lost, but injury was inflicted in proportion to their force. Hence, it must needs follow that the sick man sometimes encountered, in addition to his malady, assaults not less real because well meant. In this respect, certainly, we have evidence of progress. We are satisfied that we do not err in saying that the most judicious practitioners of the present day accept the following maxims of that eminently conservative physician, Chomel: *first*, that we are not so much to treat diseases, as patients affected with disease; and *second*, that not to do harm, is no less an object of treatment than to do good.

In defining conservative medicine, we have seen that it expresses a characteristic of the improvements in medical practice during the last twenty-five years. Let us now direct our attention to illustrations afforded by some of the different classes of remedial measures. And, first of all, blood-letting suggests itself. How great the change as regards this remedy! Twenty-five years ago it was employed as if it were an innocuous remedy. Practitioners thought much more of the risk of not resorting to it when it was needed, than of the evils of its being needlessly resorted to. Hence, they often acted on the rule inculcated by a medical writer, viz., when in doubt use the lancet. How different the rule of treatment now! Few practitioners of the present day would resort to this remedy in any case in which its appropriateness seemed to them questionable. Why not? Because it has been ascertained to be a spoliative remedy. It causes a disproportionate loss of the corpuscular elements of the blood, which are slowly regenerated. These corpuscular elements are already deficient in many diseases. In short, anæmia and its pathological relations were very imperfectly understood a quarter of a century ago. It is clear now to every one that, if not indicated, blood-letting should never be employed. This simple statement explains, in a great measure, the comparative disuse of blood-letting. The great question now is, whether it is a remedy called for more or less frequently in the management of certain diseases, chiefly the acute inflammations. I do not propose to enter here into a discussion of this question. This much may be said: clinical observation, which is alone competent to settle the question, has shown that it is a remedy not called for so often or to so great an extent in acute inflammations as was supposed but a few years ago. A single incidental remark with respect to blood-letting, and it is one which will apply to other remedies: In determining its influence for good or evil by means of clinical observation, it is not enough to take into account the ratio of recoveries, and the duration of cases of disease. Blood-letting may not increase the mortality from a disease, nor protract its continuance, and, yet, prove injurious. The injury may be manifest only in the slowness of convalescence and the impaired condition of the system after recovery.

Cathartics were prescribed a quarter of a century ago much more generally and to a much greater extent than at the present time. In fact, purgation was considered as rarely out of place, whatever might be the nature or seat of the disease. This harmonized with the notion that very many diseases originated in, and nearly all were liable to be perpetuated by, causes acting within the alimentary canal. Abernethy's views of the constitutional origin of local diseases were generally received and acted upon, and with him the constitution and the bowels were almost convertible terms, constitutional treatment consisting in the nightly blue pill and the morning black draught. The great Sir Astley Cooper quoted with approbation the quaint saying of an old Scotch doctor, who declared that fear of God and keeping the bowels open were the chief requisites of duty for safety in this world and the world to come! The importance of purgation became deeply rooted in popular sentiment. Cathartic pills or potions were considered indispensable in every household, and it would hardly express the frequency with which they were used, to say that family devotions were far less common. These were the days when, as Stokes remarks, more truly than chastely, doctors seemed to have always in their minds "a cathartic and a potfull of fæces." In this day, when a change has taken place as respects the employment of purgatives, physicians suffer from the fact that it takes a long time to eradicate a firmly fixed popular notion. Not only do we find it often embarrassing to reconcile patients to a different practice, but we are expected to inquire into, and carefully examine daily, by sight and smell, the excretions of patients, when we might otherwise consult our comfort (to say nothing of dignity) by dispensing with this exercise of the senses. The objects for cathartics, as now considered, are comparatively few, consisting chiefly in the removal of constipation, and their hydragogue operation in dropsy. They are no longer given as a matter of course, without definite indications. As perturbatory and debilitating agents, they cannot but do harm if not required, and their frequent repetition conflicts with nutrition, and thereby with sustaining measures of treatment. The change, as respects this class of remedies, thus illustrates the principle of conservatism.

It is needless to remind the reader familiar with the practice current twenty-five years ago, of the frequency with which emetics were employed. Of morbid causes referred to the alimentary canal, a large share were supposed to exist in the *primæ viæ*—an expression then often used by writers and in common parlance. The same notion taken up by the public was conveyed by the homely expression "foulness of the stomach." Emetics were prescribed by physicians to remove saburral matters, and vomiting desired by patients as a cleansing operation. Severe and prolonged vomiting by lobelia, in conjunction with the vapor bath, constituted the Thompsonian practice, which, in certain parts of our country, for several years, was considerably patronized. At the present time, emesis, irrespective of cases of poisoning and over-repletion, is rarely produced, excepting as incidental to the use of remedies not prescribed for that purpose, such as the nauseant sedatives, colchicum, veratrum viride, etc. What would be thought of a practitioner now who treated cases of phthisis with emetics repeated almost daily! Yet, within the memories of physicians of twenty-five years standing, this practice has been advocated, and, to some extent, adopted. The progress of medical conservatism has led to the abandonment of emetics, as perturbatory and debilitating agents, excepting in the rare instances in which they subserve an explicit purpose.

The practice of the present time presents a striking contrast with that twenty-five years ago, as regards the use of counter-irritant applications. The physician whose professional career has already extended over that period, is sometimes reminded of the severe measures then in vogue, by the exhibition of indelible scars on the bodies of his old patients. He is not likely now to contemplate these traces of his former vigorous practice with lively gratification. Blisters, sometimes applied successively over the same space, and not diminutive in size, tartar emetic ointment and plasters, issues, the moxa, etc., were considered as among the most efficient of the means of influencing the cure of a host of local affections. How much less frequently are they now used, and, when counter-irritation is deemed advisable, how much milder are the applications chosen! Physicians were strongly impressed with the belief that local affections were often removed by revulsion. They accepted the doctrine of Hunter, that two diseases rarely concur, and hence, that an artificial disease is likely to effect a cure by a process of displacement. Not only has this doctrine been disproved by pathological researches, but these have shown a large number of the local diseases formerly regarded as primary, to be the secondary or tertiary effects of morbid conditions then unknown. Bright's disease had not been discovered, and its multitudinous pathological conquences were, of course, unintelligible. In those days solidism prevailed, and hæmatology has been since created. Physicians made no account of blood poisons, and the old humoral notions of coction and fermentation had not been revived under the modern but equally indefinite garb of catalysis. Mr. Farr had not invented the name Zymosis, a name expressive of our ignorance rather than conveying any precise knowledge, but, nevertheless, significant of a wide and most important leap from the doctrine of solidism; or, in other words, of a passage backward, guided by the light of modern science, to humoralism, which, as Rokitansky remarks, is simply a requisition of common sense. This change in pathological views, in conjunction with clinical observation, has led physicians to distrust, more and more, the value of counter-irritant applications, and, at all events, to conclude that severe revulsive measures are rarely called for; hence, the change in practice is in comformity to the principle of conservatism.

The contrast as regards the use of mercury affords a signal instance of progressive change. The remarkable efficacy of this remedy in certain affections naturally led to the expectation of its utility in many diseases. Mercurialization being a disease, it accorded with the current belief of the incompatibility of different affections, to suppose that it displaced other diseases. It was considered as *par excellence* an *alterative* remedy; and what a latitude for imagined results was afforded by that title! Moreover, its supposed special action on the liver accorded with the notion that the secretion of bile had much to do with morbid phenomena. The relief or prevention of portal congestion was incidental to its hepatic effects. It lessened exudations; it promoted the absorption of morbid products; it altered the secretions; it dispelled local engorgements, and, by exciting stomatitis, it acted by way of revulsion. Waiving here, as in the other instances, discussion of the actual value of this remedy, the extravagance of the views formerly entertained is now sufficiently evident. The statements of those who have made war upon this article of the materia medica, and the popular prejudices thereby produced, are equally, or still more, extravagant; but it is a remedy potent for harm when inappropriate, as it is powerful for good when indicated; and, therefore, the great change that has taken place as regards its use exemplifies conservatism.

These examples are sufficient to show how conservative medicine is illustrated by recent improvements as regards the employment of particular therapeutic measures. They furnish evidence of immense progress in practical medicine. Let not this statement be misunderstood. The improvements which have been noticed consist in the restricted use of blood-letting, cathartics, emetics, counter-irritants, and mercurials. Does the restricted use of these measures detract from their real therapeutic value? Not at all. Medicine has, by no means, repudiated them. She employs them with better judgment and discrimination; thus availing herself of the good they can accomplish, she escapes the evils arising from their injudicious and indiscriminate use.

If we look at the progress of medicine during the last quarter of a century, from another point of view, we find additional examples of conservatism. Regarding it exclusively from the point of view already taken, it appears that, in proportion as the practice of medicine has improved, reliance on certain active or heroic measures of treatment has diminished. This is true, but it is not the whole truth. Some measures are employed with much more freedom now than a few years ago. The use of opium and alcoholic stimulants, in certain diseases, affords the most striking illustrations of this truth. These instances also exemplify the principle of conservatism. Opium and alcohol, in excessive doses, occasion immediate disorder, of more or less gravity, and may destroy life. But given so as not to incur any risk of these effects, they do not conflict with conservatism, because their operation is transient, and, unless their use be continued, they do not leave behind them damaging effects. Given in quantities which are comfortably borne, they certainly do not impair the vital forces by perturbation, by loss of fluids, by affecting the constitution of the blood, or by inducing local changes, as do the measures previously noticed. This statement, of course, has nothing to do with the ulterior consequences, moral and physical, of intemperance or opium eating. Here, too, as in other instances, discussion of the *modus operandi* of remedies is waived. Most physicians will agree in the statement that, when indicated as remedies, opium and alcohol sustain the vital forces. In this respect they are positively conservative. But a point of distinction is, when not indicated, if given within certain limits, and not continued, they are neither spoliative, exhausting, disturbing, nor disorganizing, as are various other measures, and, therefore, not, like the latter, even then, antagonistical to conservative medicine.

The contrast between the practice of medicine now and twenty-five years ago is not less marked, as regards the use of opium and alcohol, than as regards the restricted employment of other measures. Let the practitioner, who has seen service for a quarter of a century, consider what a responsibility he would once have taken in treating cases of pneumonia with brandy and opium, to say nothing of the continued fevers. The wonderful tolerance of these remedies in certain cases of disease is a recent discovery. Let the same practitioner consider whether he would once have ventured on a hundred grains or more of opium *per diem* in a case of peritonitis, or grain doses of the sulphate of morphia hourly, continued for several days, in a case of dysentery. Let him consider whether, at the commencement of his career, with the fulminations of Broussais on incendiary practice resounding in his ears, he ever dreamed of the propriety of giving quarts of spirit daily to fever patients, and of finding the frequency of the pulse diminished, and the mind become more clear under this heavy stimulation !

If we turn from remedial measures to dietetics, we find that the improvement which has taken place in practice contributes to the illustration of conservative medicine. In fact, conservatism is, perhaps, not less conspicuous in the contrast as respects the diet of the sick than in any other point of view. In cases of fever, and all acute diseases, twenty-five years ago, it was generally deemed an essential part of the treatment to withhold alimentary supplies. It was a frequent saying to patients who craved food, that to allow it would be to nourish the disease. In chronic affections, too, the diet was usually much restricted. It was believed that a large majority of diseases were attributable, directly, to dietetic imprudences, and that the over-ingestion of food, during the progress of diseases, was, of all indiscretions, the most prolific of evil. Physicians seemed to lose sight of the plain fact that the vital powers must languish in proportion as the alimentary supplies fall below the wants of the system, and that death may be produced by starvation in disease as well as in health. At the present time, a nutritious diet is considered as highly important in the management of fevers, as well as in diseases which tend to destroy life by exhaustion, and most physicians appreciate the importance of keeping the body well nourished in chronic affections.

Incidentally a point for remark is here suggested. Twenty-five years ago disorders of digestion, grouped under the name dyspepsia, were extremely frequent. Dyspepsia was the popular malady of the day. The number of dyspeptics, of late years, has greatly diminished. The malady is comparatively infrequent. Why is this ? I believe it to be explained, in a great measure, by the fact that in the matter of eating, instinct has regained its rightful supremacy. We do not hear so much now, as then, of the liabilities to dietetic errors. Physicians are not so ready to attribute diseases to some imprudence at the table. The subject is not brought to the minds of the people by means of conversation, popular books on diet, public lectures and sermons. The healthy man no longer sits down to dinner with fear and trembling, lest he should eat too much, or indulge in improper articles of food. There are fewer patients who hold to the fanatical notion, that moral and physical health requires the demand of the system for food in sufficient quantity and variety, as expressed by hunger and appetite, to be resisted; and that the welfare of body and mind is promoted by living on a poor and insufficient diet. We rarely, now-a-days, hear the injunction, which was once impressed upon all who would preserve health, to adopt the habit of always rising from the table hungry. Nature and common sense have triumphed over these absurd ideas, and, among other advantages, dyspeptic ailments, which formerly tormented so many persons, have wonderfully diminished.

Recurring to the definition of conservatism in medicine, it suffices to say that it means the preservation of the vital forces. It is a principle in medical practice, covering everything which prevents impairment of, or tends to develop and sustain, the powers of life. The terms "vital forces" and "powers of life," although they are not readily explained, have a practical meaning, which is well enough understood, and it is unnecessary to enter into an explanation of them. It has been the object, in the foregoing pages, to give an exposition of conservative medicine, and to show that conservatism, in the sense in which the term is now used, is a distinguishing feature of medical practice at the present time, as contrasted with the practice which prevailed twenty-five years ago. The development and adoption of this principle have been seen to be results of the progress of medical knowledge, and the circumstances which seem especially to mark the beginning of the changes illustrating the principle are, abandonment of attempts to reduce the practice of medicine to a system, after the failure of the latest, viz., Broussaisism, and the study of the natural history of diseases, as inaugurated by Louis. It is by no means, however, intended to ignore the fact that the cultivation of all the branches of medical knowledge has powerfully co-operated to the same end. The changes which have taken place during the last quarter of a century have not been due to a prior recognition of the principle of conservatism, but now that the changes have occurred, we find conservatism to be common alike to all, binding them together, and constituting their most striking characteristic. Having reached the principle thus analytically, are we not bound to recognize it as a fixed principle of medical practice, and one possessing great practical importance? Assuming it to be such, the remainder of this article will be devoted to its applications in the management of different forms of disease. And, first, let us consider the application of conservatism to the treatment of patients with inflammatory affections.

Theoretical views led to the measures called *antiphlogistic* in cases of inflammation. These measures, consisting of general and local blood-lettings, cathartics, and rigid or restricted diet, were considered as antagonizing the state of inflammation; not unfrequently arresting its progress, and, when not successful in this end, diminishing its severity, limiting its morbid effects, and abridging its duration. As already remarked, the injury which these measures are capable of doing was overlooked, and, on the other hand, all will admit that their efficacy, in effecting the objects just stated, was greatly over-estimated. Clinical experience has shown that we cannot rely upon these measures to arrest the progress of inflammation. Admitting the possibility or probability of success in a small proportion of cases, we are not justified in exposing patients to the injury produced if these measures do not succeed, when the chances are few that they will prove successful. This statement expresses a rule of conservatism applicable to all potent measures employed in any disease as abortive measures of treatment. Measures not impairing the vital forces are allowable, even when the probability of success is small. Opium, for example, is admissible as an abortive remedy when blood-letting is clearly inadmissible. But measures which, if not successful, will do harm, are only to be resorted to when the chances of success preponderate over those of failure. Conservatism, therefore, does not justify the employment of the antiphlogistic treatment with a view to the arrest of inflammation, without taking the ground that they invariably fail.

Clinical experience has rendered it doubtful whether the antiphlogistic treatment exerts much effect on the intensity of inflammation, its results, or its duration. Conservatism, therefore, dictates a careful weighing of the evils of the treatment against the chances of its usefulness as regards these objects.

It is not settled by experience that this treatment, carried to a greater or less extent, is always in no measure efficacious. Hence, there is room for difference of opinion, and the practice of different physicians will differ. The discriminating practitioner, who, although satisfied of the evils of the indiscriminate employment of antiphlogistic measures, believes in their ability, if judiciously employed, will be guided in withholding or resorting to them, by the circumstances belonging to individual cases. And here it is that his practical knowledge, judgment, and tact are brought to bear on the management of inflammatory affections. Conservatism will dictate to such a practitioner not to employ blood-letting, etc., when the inflammatory affection, from its seat and degree of intensity, involves no danger, and when there is reason to suppose that it may pass through its course favorably, without active interference. Conservatism will dictate the same policy when all the local results to be expected from the progress of inflammation have already taken place, and the restorative processes only remain—a condition illustrated by the second stage of pneumonia, when all the exudation that is to occur has occurred, and the recovery involves only the absorption of the morbid deposit. Conservatism will dictate the same line of conduct in all cases of disease in which more danger is to be expected from failure of the powers of life, than from lesions incident to the local affection.

The value of therapeutic agencies is, of course, to be determined by experience. Developments in the progress of pathology, however, contribute to our knowledge of therapeutics, not only by giving direction to clinical observation, but by harmonizing with the conclusions drawn from the latter. It is interesting to note the consistency of the practical views now generally held as regards antiphlogistic measures, with late developments respecting the origin of certain inflammations. Inflammations not traumatic were formerly considered, and are now often called spontaneous. We may use this term conventionally as distinguishing a local disease not referable to any obvious local cause, but, strictly, it is an absurdity to say that any disease is spontaneous. Every local affection must involve an adequate morbific agency acting on the part affected. It is true that our present knowledge does not enable us generally to appreciate the nature, sources, and the *modus agendi* of the proximate causes of inflammatory affections, but we have acquired, of late years, some information important in itself as a basis for analogical reasoning. Clinical observation has shown that the accumulation of urea in the blood is apt to lead to inflammation of serous structures. This we know, and it is a rational supposition that urea (or the products of its decomposition) induces inflammation, by acting directly on these structures. There are sufficient grounds for believing that the local inflammations occurring in gout and rheumatism are due to the local action of a *materies morbi* in the blood, perhaps the uric acid in the former, and the lactic acid in the latter of these diseases. Reasoning by analogy we may expect with considerable confidence that future researches will show the so-called spontaneous inflammations generally to be produced in a similar manner. And with this view of their production, we should rationally expect great results, not so much from the antiphlogistic treatment as from measures addressed to the morbid conditions of the blood which underlie the local manifestations of disease. To ascertain these morbid conditions in different diseases, to prevent the introduction or accumulation of morbific material in the blood, to neutralize the poisonous properties of this material, by causing the promotion of innocuous combinations, prevent the organico-chemical changes which its presence induces, (catalysis,) or to eliminate it through the emanations of the body,—these are the great objects of therapeutics at the present day, which harmonize with the late revelations of pathology. Without stopping to inquire how far these objects have been obtained, it is to be remarked that they are obviously conservative, involving, as they do, protection against internal agencies inimical to life and health.

Conservative medicine thus dictates, in inflammatory affections, proper discrimination in the employment of the so-called antiphlogistic measures, which, if failing to exert a controlling influence, are necessarily hurtful, and, it may be, destructive, by impairing the vital forces. It also dictates the judicious use of remedies addressed to the internal causative conditions pertaining to the blood, so far as our present knowledge extends into these most important provinces of pathology and therapeutics. But this is not all. Conservatism often demands that the vital powers shall be sustained. Sustaining measures of treatment, practically considered, consist of tonics, alcoholic stimulants, and nutritious diet. We will not inquire as to the rationale of the operation of these measures. Suffice it to say, clinical experience shows abundantly that they lessen the degree to which the vital forces would otherwise be impaired by disease, and may prevent a fatal termination of disease by exhaustion. I have already admitted that the phrase "powers" or "forces of life" is metaphorical. Life is not an entity. But with a fair understanding that this personification of a combination of conditions, as yet but imperfectly understood, is merely for convenience, it is unobjectionable. The powers or forces of life enable the system to bear up under disease, to resist it successfully, and recover from it. On the other hand, we may say that disease destroys by overcoming the powers of life, whenever death takes place by asthenia or exhaustion. Every sagacious practitioner knows that certain symptoms, no matter with what disease they are associated, denote failure of the vital powers, or inability to resist disease. He estimates the amount of danger by these symptoms, among which those referable to the circulation are especially important. He often bases his prognosis far more on these symptoms than on the nature and extent of the local affection. Every practitioner knows that an inflammation, the same in all respects, so far as the local affection is concerned, in different persons, affects the vital forces differently. Take, for example, pneumonia, extending over the same space, and inducing an equal amount of changes, which physical exploration enables us to determine with accuracy: one patient manifests little disturbance of the system, and no symptoms denoting danger, while another patient will succumb to the disease. Every practitioner knows that some persons, who, in health, present no evidence of a lack of vigor, have very little ability to resist severe disease. They are quickly destroyed by affections which other persons readily endure, and endure perhaps without much inconvenience. Of course, these facts are explicable, but not with our present knowledge, and, until explained, it answers to refer them to differences as regards the vital powers or forces. They are facts of not a little practical importance.

Conservatism dictates sustaining treatment in any inflammatory affection whenever the symptoms denote failure of the vital powers, whether the period be early or late in the course of the affection. This treatment is to be pursued vigorously in proportion to the rapidity of the failure and the amount already taken place. It is important in all dangerous affections to watch for the first evidence of failure, and to lose no time in resorting to supporting measures. Such is the influence of traditional ideas, that these measures are frequently delayed from a timidity which experience is sure to remove. It is far wiser to enter on the use of tonics, stimulants, and a nutritious diet too early, or when not required, than to incur risk of delay, or their omission when required. In the one case, the liability of harm is small, but in the latter, lost time, which cannot be regained, may have been of immense importance to the patient. So far from incurring risk of damage from delay, the wise practitioner will anticipate the indications for support, and forestall the failure which he knows would otherwise occur. Physicians, however devoted to the antiphlogistic treatment of inflammations, have generally recognized the importance of supporting measures to "obviate tendency to death." When the flame of life is reduced to a glimmer, they would prevent it, if possible, from going out. Does not common sense teach that measures which may prove serviceable under these circumstances, would have proved much more so when the danger was less imminent? Is it not better policy to endeavor to keep the lamp of life burning brightly, than to depend on efforts to restore the flame when nearly extinguished? In cases involving danger to life, the importance of sustaining treatment is to be measured by these questions: Is the chief danger due to failure of the vital powers, and how great is the danger from this source? In cases not involving danger to life, the importance of support has reference to the duration of the disease, the rapidity of convalescence, and the condition of the recovery. The advantages derived from the proper application of conservatism, as regards sustaining treatment, in all operations, by no means consist exclusively in a reduced rate of mortality, but also in a speedy and rapid convalescence, and in the completeness of the restoration to health.

These remarks have had reference more especially to acute inflammations. Chronic inflammation affecting an important part may continue for a greater or less period, and recovery finally be complete; but during its continuance the powers of life are more or less impaired. It may destroy life by leading to incurable lesions, or by its protracted duration; in either case death usually taking place by slow asthenia. Under all circumstances, the affection is less likely to be prolonged, serious changes of structure are less likely to take place, and a fatal termination is postponed in proportion as the vital powers are preserved. Conservatism, therefore, dictates not measures to reduce, but those which sustain the powers of life in chronic inflammations. It dictates measures to develop appetite, and improve the digestive processes, abundant nutritive supplies, and, in short, the remedies and hygienic means which invigorate and strengthen the body. The "building up" treatment, as it is significantly called, has contributed largely to the more successful management of chronic affections since the days of Broussaisism. Some of the most striking examples of the efficacy of this treatment which I have seen have been cases of chronic pleurisy, in which speedy and progressive improvement followed directly the substitution of this treatment for measures opposed to the principle of conservatism. These examples are the more satisfactory because, by means of physical signs, the improvement within the chest was accurately determined at the same time that the local and general symptoms denoted a favorable charge. Certain cutaneous inflammations, and cases of ophthalmia, the parts in these affections being open to inspection, also afford examples not less striking.

Conservatism has been practically more fully applied to the management of essential fevers than of inflammatory affections. Since nearly all pathologists have admitted the essentiality of fever, and since physicians have ceased to agree with Southwood Smith in regarding inflammation as an almost constant concomitant and the chief source of danger in fever, the importance of preserving and sustaining the powers of life has been more and more appreciated, and, at the present moment, with the most intelligent practitioners, these are the leading objects in the treatment. In this remark I refer especially to fevers having a self-limited career, and not arrested by abortive measures. The periodical fevers are controllable by remedies having a special efficacy. These remedies are conservative, acting in an imperceptible manner, and, given within proper limits, producing no destructive or injurious effects even if not indicated. It is a curious fact that the fevers which we are able to arrest with great certainty, *i.e.* the periodical fevers, continue indefinitely if not arrested, and return, sooner or later, and more or less frequently, in the majority of cases, whereas the fevers which we cannot arrest with any certainty, if at all, *i.e.* the continued fevers, the eruptive, and yellow fever, have a fixed duration, and, as a rule, are experienced only once. It is not without the bounds of a reasonable expectation that the means of arresting the last-named fevers will hereafter be discovered. Reasoning by analogy, and from the pathological views now generally entertained, the means for this end must act by neutralizing a morbid material in the blood, or effecting its elimination, acting, therefore, in accordance with the principle of conservatism.

Tonic remedies, alcoholic stimulants, and nutritious diet are the measures for maintaining the vital forces during the course of the essential fevers. The importance of these measures is now so generally admitted as hardly to require argument or advocacy. The only questions for discussion relate to circumstances indicating their employment, the extent to which they are to be carried, and various details connected with their use. The discussion of these questions does not fall within the scope of this article. I may be indulged, however, in a few remarks on some interesting points connected with the subject.

One of these is the wonderful tolerance of alcoholic stimulants in certain cases of fever. Examples have been of late so often repeated, and are so generally familiar, that they need not be cited. How much at variance are the effects of pints or even quarts of spirit, given daily, with those produced in health! And how fully does this fact, as well as analogous facts relating to the action of opium and other remedies, illustrate the liability to error in judging of the operation of therapeutic measures in disease from experimental observations in healthy persons! How surprised, but a few years ago, would have been the therapeutist if told that the action of alcohol, under certain morbid conditions, is in fact sedative; in other words, that, in certain cases of typhus and typhoid fever, two or three ounces of spirit given hourly lessen the frequency of the pulse, diminish the heat of skin, and render the mind more clear! But the past history of medicine shows a tendency to push prevailing ideas to an extreme, against which the prudent physician should endeavor to guard himself. There is danger now of carrying the use of alcohol to an injudicious and dangerous extent. The principle of conservatism should be the guide. The object is to sustain the vital forces. The tolerance is in proportion to the need of this sustaining agent. If it be used excessively in all cases, without discrimination, it will sometimes do harm, and life may be destroyed by alcoholic poisoning. We have already seen that, within certain limits, alcohol is eminently a conservative remedy, because even when not indicated, it is not destructive, and its operation is transient, but beyond certain limits its effects may be poisonous, provided it does not fulfill indications showing that the system is tolerant of quantities which would be dangerous in health. Let the indications, then, in individual cases, be carefully observed, and let the effects be carefully noticed, so as not to violate, but conform to the rule of conservatism.

Some interesting points are connected with the dietetic management in cases of fever. In perfect health the wants of the system for alimentary supplies are expressed by hunger and appetite. Common observation, however, teaches us that these sensations are not essential as prerequisites to digestion and nutrition. Almost every one has experienced a state, certainly abnormal but not dependent on any well-defined disease, and not interfering with the usual habits of mental and physical activity, in which food is taken habitually for a greater or less period without hunger or appetite, and nevertheless properly assimilated. Intense mental preoccupation and persisting depressing emotions may involve such a state. During the career of fevers, usually, hunger and appetite are wanting, but it is not to be inferred therefrom that the ability to appropriate nutriment is lost. Some have reasoned that the absence of the desire for food is always evidence of its not being needed, and a comparison has been made between the morbid condition, in this regard, in the essential fevers, and the natural state of hybernation. But the analogy holds good only as respects the disinclination for food. In hybernation, the respirations, the heart's action, muscular movements, and the functional exercise of all the organs, are reduced to the lowest point compatible with the preservation of life. In fever, the respirations are far oftener increased than diminished in frequency, and more oxygen enters the system than in health; the heart beats with unwonted frequency, muscular action is not wanting, and in the more frequent respiratory movements it is above the healthy standard; the mental faculties are sometimes morbidly active, and, from the absence of sleep, often more continuously so than in health; calorification is increased, and various functions of the body manifest disordered activity. It seems sufficiently clear that no practical inferences are to be drawn from a comparison between the arrest of hunger and appetite in fever, and the suspension of these sensations in hybernation. In hybernation the system has no need for alimentary supplies, and, hence, there is no physiological expression of the want of them. In fever the morbid conditions prevent the feeling of this want, although the need of alimentary supplies continues.

The correctness of the statement just made rests on clinical observation. Patients with fever, taking food without inclination and even with repugnance, retain it, and no disturbance is produced by its ingestion; the fecal evacuations may present a normal appearance, and, in some cases in which a nutritious diet has been entered upon after the disease has existed for some time, there is an evident increase of muscular strength, although the career of the fever continues. These are clinical facts. And the conclusion is, digestion and nutrition are not incompatible with the state of fever, although hunger and appetite may be wanting. The faculty of perceiving these sensations is impaired or lost in consequence of the morbid condition of the nervous system, and, hence, they cease for the time to express the demands of the system. The perceptions are often so blunted that the mind takes no cognizance of other wants of the system. The urine is allowed to accumulate in the bladder, and, with the tongue desiccated, the patient manifests no desire for drink. Fatigue from lying continuously in the same position is not complained of. Local complications of the disease are not accompanied by pain. Under these circumstances, it is consistent that the sensations of hunger and appetite should not be experienced. The perceptive faculties, however, sometimes are not so much impaired as they appear to be. Desires and feelings may not be manifested from an extreme reluctance to make any exertion. Thus, patients not unfrequently drink with avidity when the cup is brought to their lips, who make no complaint of thirst; and in some cases, food, when presented, is also taken with relish.

In sustaining the powers of life in fevers, then, (and also in certain other diseases,) the physician is not to be restrained by the absence of hunger and appetite. He is to act with reference to the wants of the system by endeavoring to secure the ingestion of food, concentrated, containing the necessary variety of alimentary principles, and ample in quantity. Here, too, as with regard to alcoholic stimulants, it is far better to begin earlier than is needed, than run any risk of delay, and to give more aliment than is required than not enough. An appreciation of the importance of alimentation in fevers is among the most important of the recent improvements in practice which exemplify the spirit of conservatism. But in all acute diseases, whenever the chief end of treatment is to support the powers of life, a nutritious diet is essentially important, and the same rules with regard to dietetic management are alike applicable.

It will suffice to notice the application of conservatism to those chronic affections collectively which destroy by gradual inroads upon the powers of life. In this class are grouped such affections as carcinoma, tuberculosis, chronic dysentery, cirrhosis, and Bright's disease. It is sufficiently clear that, with a view to the prolongation of life, when recovery is not expected, the great object is to retard, as much as possible, the failure of the vital forces. If we cannot "build up," we may do much to delay the progress of destruction. Evident as this is, it is not sufficiently appreciated by all practitioners.

Patients affected with incurable diseases are too often abandoned to merely palliative remedies, the fatal issue being considered as merely a question of time, and, therefore, not of much importance. This question of time, however, may be highly important to patients and their friends. To aid in the cure of diseases is, undoubtedly, the first aim of the physician; and next to this, when a cure is not to be effected, comes the prolongation of life, with health more or less impaired. The last of the grand objects of practice are palliation and euthanasia.

In the management of any incurable affection, conservatism dictates the measures which, in general terms, contribute to keep the body in the best possible condition compatible with the continuance or progress of the disease. In this way not only the inroads of the disease on the powers of life, but the destructive lesions in the parts affected, are often stayed. It may be assumed to be a rule in pathology that a local affection involving structural changes is less likely to progress with rapidity, the closer the approximation to health in all other respects. The practice which conservatism dictates in such cases is in accordance with this rule. An incurable lesion is sometimes so completely held in abeyance, and the system is rendered so tolerant of its continuance, that life may be preserved indefinitely, although a vital organ be affected. We meet with cases in which depositions of tubercle and carcinoma remain for a long period non-progressive and nearly innocuous. The conservative practice, moreover, favors those retrogressive changes by which even the diseases just named may eventuate in cure.

To consider the measures for keeping the body in the best possible condition, would be to enter on a large but immensely important domain of practical medicine. I must content myself with saying that they consist, first, of a nutritious diet; next, of remedies to strengthen and invigorate; and last, of hygienic influences directed to the same end. The hygienic influences comprise exercise and everything relating to regimen, change of climate, mental diversion and encouragement—in short, whatever can be brought to bear favorably upon the welfare and vigor of the system. The hygienic is certainly not inferior in importance to the medical treatment, and here it is that the judgment and tact of the successful practitioner are especially brought into requisition.

A comparison of cases of pulmonary tuberculosis now and twenty-five years ago, illustrates the importance of the practical views just presented. The management of this disease twenty-five years ago was certainly not in accordance with the principle of conservatism. The measures employed, medicinal and hygienic, were, indeed, directly opposed to this principle. The antiphlogistic system of treatment was often adopted, under the belief that inflammation was the most important element of the local affection. Blood-letting, cathartics, mercurialization, severe counter-irritation, were considered as remedial, and to these were conjoined low diet and confinement within doors. Now, pulmonary tuberculosis is not cured in the majority of cases, although it is not incurable, and there is reason to believe that the proportion of cures is considerably larger than under the treatment just referred to. But, directing attention to the incurable cases, under the plan of treatment generally pursued at the present time, which is eminently conservative, how striking the contrast! Formerly, the instances of rapid progress of the disease were more numerous, and it almost invariably advanced with a steady march, rarely occupying many months in completing its fatal career. Patients were usually confined to the bed for weeks before death, lingering on the borders of the grave, suffering from extreme debility, bed-sores, aphthæ, and colliquative diarrhœa. It was difficult to conceive of a picture more distressing and repulsive than that of an unfortunate being in the last stage of consumption. Conservatism has done much toward ameliorating the condition of consumptives, even when it is hopeless as regards recovery. Cases of so-called galloping consumption are less frequent. Life is not unfrequently prolonged and made comparatively comfortable for years. It is not uncommon to meet with instances of a considerable deposit of tubercle remaining quiescent or progressing very slowly, and the patient able to engage in the active occupations and enjoyments of life. Even when the disease is progressing to a fatal termination, the strength is usually so far preserved that a bed-ridden consumptive is now rarely seen, and it is not uncommon for patients to be out of doors almost up to the hour of their death. I appeal to those whose medical experience has extended over a quarter of a century for the truthfulness of this comparison.

In concluding these fragmentary remarks, let it be borne in mind that, important as is conservatism in medical practice, it is by no means inconsistent with the employment of efficient therapeutic agencies in the management of diseases. The conservative surgeon does not hesitate to use the knife and dismember the body, when convinced that thereby he may save life. So the conservative physician resorts without hesitation to his potential remedies—not less potent for good or evil than the scalpel—whenever he sees clearly that they will contribute to the safety and welfare of the patient.

